# Frequency- and spike-timing-dependent mitochondrial Ca^2+^ signaling regulates the metabolic rate and synaptic efficacy in cortical neurons

**DOI:** 10.7554/eLife.74606

**Published:** 2022-02-22

**Authors:** Ohad Stoler, Alexandra Stavsky, Yana Khrapunsky, Israel Melamed, Grace Stutzmann, Daniel Gitler, Israel Sekler, Ilya Fleidervish

**Affiliations:** 1 Department of Physiology and Cell Biology, Faculty of Health Sciences and Zlotowski Center for Neuroscience, Ben–Gurion University of the Negev Beer Sheva Israel; 2 Department of Neurosurgery, Faculty of Health Sciences and Zlotowski Center for Neuroscience, Ben–Gurion University of the Negev Beer Sheva Israel; 3 Rosalind Franklin University of Medicine and Science, Chicago Medical School, Center for Neurodegenerative Disease and Therapeutics North Chicago United States; Brandeis University United States; Stanford University School of Medicine United States

**Keywords:** cortical pyramidal neuron, spike-timing-dependent plasticity, mitochondria, Ca2+ signaling, axon, dendrite, Mouse

## Abstract

Mitochondrial activity is crucial for the plasticity of central synapses, but how the firing pattern of pre- and postsynaptic neurons affects the mitochondria remains elusive. We recorded changes in the fluorescence of cytosolic and mitochondrial Ca^2+^ indicators in cell bodies, axons, and dendrites of cortical pyramidal neurons in mouse brain slices while evoking pre- and postsynaptic spikes. Postsynaptic spike firing elicited fast mitochondrial Ca^2+^ responses that were about threefold larger in the somas and apical dendrites than in basal dendrites and axons. The amplitude of these responses and metabolic activity were extremely sensitive to the firing frequency. Furthermore, while an EPSP alone caused no detectable Ca^2+^ elevation in the dendritic mitochondria, the coincidence of EPSP with a backpropagating spike produced prominent, highly localized mitochondrial Ca^2+^ hotspots. Our results indicate that mitochondria decode the spike firing frequency and the Hebbian temporal coincidences into the Ca^2+^ signals, which are further translated into the metabolic output and most probably lead to long-term changes in synaptic efficacy.

## Introduction

For the neuronal circuit to function properly, the energy demand in all compartments of the individual neurons needs to be precisely matched by local, primarily mitochondrial (Celsi et al., 2009; Rangaraju et al., 2019), ATP production. During periods of enhanced neuronal activity, mitochondria accelerate ATP production by allowing the cytosolic Ca^2+^ elevations to propagate into the mitochondrial matrix (Ashrafi et al., 2020; Díaz-García et al., 2021). When cytosolic [Ca^2+^]_i_ rises, Ca^2+^ ions, powered by the steep mitochondrial membrane potential, flow into the mitochondrial matrix via the Ca^2+^ uniporter MCU (Baughman et al., 2011; De Stefani et al., 2011), and are then extruded back into the cytosol by the mitochondrial Na^+^/Ca^2+^ antiporter NCLX (Palty et al., 2010). The mitochondrial Ca^2+^ elevation increases ATP production by activating at least three Krebs cycle enzymes (De Stefani et al., 2016; Wescott et al., 2019). When Ca^2+^ signaling is disrupted, mitochondria in presynaptic terminals fail to maintain the stable ATP concentration during enhanced activity periods (Ashrafi et al., 2020; Giorgio et al., 2013; Glancy and Balaban, 2012). The link between the distinctive types of the neuronal electrical activity, mitochondrial Ca^2+^ signaling and metabolism in other neuronal compartments is poorly understood, however.

Recent evidence using photo-uncaging of glutamate on spines of cultured hippocampal neurons indicates that dendritic mitochondria play a critical role in long-term regulation of synaptic strength (Rangaraju et al., 2019). Thus, local inhibition of the mitochondria by a phototoxic protein abolishes the synaptic plasticity in the affected dendritic segment. Under physiological conditions, the plastic changes in synaptic efficacy are believed to be controlled by the relative timing of pre- and postsynaptic action potentials (APs) (Bi and Poo, 1998; Holtmaat and Svoboda, 2009; Markram et al., 1997b). Spike-timing–dependent plasticity (STDP) relies on activation of the postsynaptic NMDA receptors, which are Ca^2+^ permeable and require both glutamate and depolarization to open (Nowak et al., 1984). Mitochondrial handling of Ca^2+^ ions is known to have a significant effect on cytosolic Ca^2+^ dynamics (Rizzuto et al., 2004; Szabadkai and Duchen, 2008). The mitochondrial depletion, however, had no effect on glutamate evoked cytosolic Ca^2+^ dynamics (Rangaraju et al., 2019), indicating that these organelles play a yet unexplored downstream role in the cascade of reactions leading to STDP.

Here, using whole-cell electrical recordings from Layer 5 pyramidal neurons in cortical slices and fluorescence imaging of cytosolic and mitochondrial Ca^2+^ indicators, we show that single or few spikes trigger rapidly rising and decaying mitochondrial Ca^2+^ elevations in all neuronal compartments, with kinetics similar to cytosolic Ca^2+^ transients. Our evidence indicates that the mitochondria’s Ca^2+^ signaling and metabolic rate depend critically on spike firing frequency. We further report that, in dendrites, the coincidence of unitary EPSP and a backpropagating action potential produces a localized [Ca^2+^]_m_ transient, which, in addition to enhancing local ATP synthesis, could play a role in STDP.

## Results

### Spike-elicited mitochondrial Ca^2+^ transients

**Figure 1.**
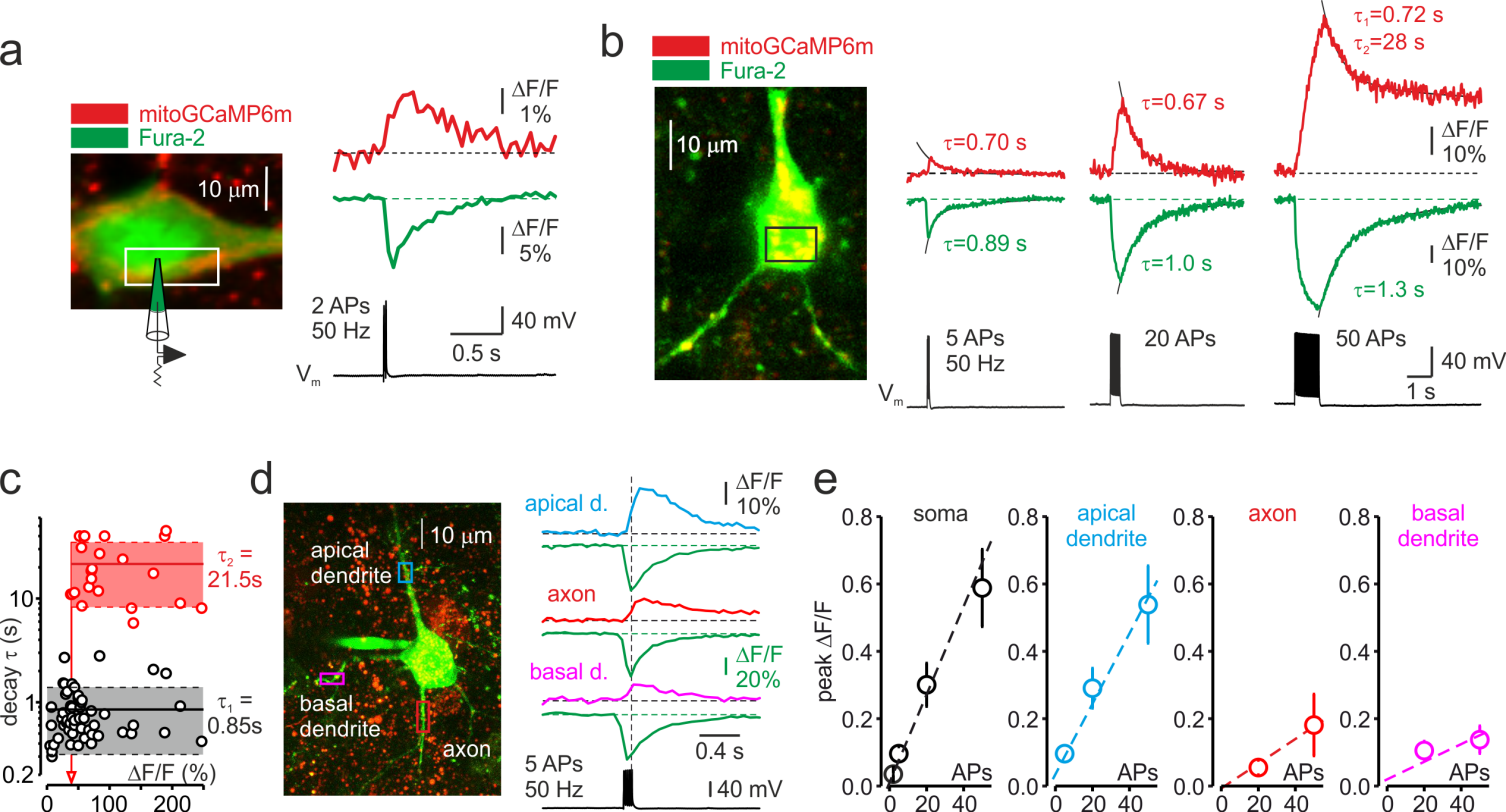
Trains of action potentials elicit mitochondrial Ca^2+^ elevations in soma and processes of L5 pyramidal neurons. (**a**) Changes in mitoGCaMP6m and Fura-2 fluorescence elicited by a train of two APs in a representative L5 pyramidal cell. *Left,* The image obtained by merging two optical sections through part of a L5 neuron at excitation wavelengths of 760 and 960 nm, eliciting the Fura-2 and mitoGCaMP6m fluorescence, respectively. *Right,* Somatic mitoGCaMP6m (red) and Fura-2 (green) ΔF/F transients elicited by a train of two APs. MitoGCaMP6m trace (red) is an ensemble average of 16 consecutive sweeps. (**b**) Small-amplitude mitoGCaMP6m transients decay rapidly. *Left*, Localization of the mitoGCaMP6m labeled mitochondria in a representative L5 neuron. The image was obtained by merging the fourteen, 1 µm thick optical sections at excitation wavelengths of 760 and 960 nm, eliciting the Fura-2 and mitoGCaMP6m fluorescence, respectively. The yellow color indicates colocalization of the Fura-2 (green) and mitoGCaMP6m (red) fluorescence, revealing the position of the mitochondria. The rectangle indicates the region of interest from which the optical sweeps were obtained. *Right*, somatic mitoGCaMP6m (red) and Fura-2 (green) transients elicited by 5, 20, and 50 APs in a cell shown on the left. Black lines are the best single or double exponential fits of the decay. Notice that after the relatively small [Ca^2+^]_m_ elevations evoked by five or 20 APs, the Ca^2+^ clearance is rapid and follows the single exponential time course (*τ* ~ 0.7 s). The decay of larger transients evoked by 50 APs is biexponential (τ_1_ of ~0.7 s and τ_2_ of ~28 s). (**c**) Decay time course of mitochondrial Ca^2+^ transients depends on their amplitude. Each dot represents a decay time constant of the mitoGCaMP6m transient (τ_1_ for monoexponential, τ_1_ and τ_2_ for bi-exponential decays) obtained in recordings from 42 neurons and plotted against its peak amplitude. Continuous lines are mean τ_1_ (n = 55, black) and τ_2_ (n = 23, red); dashed lines represent standard deviation from the mean. Arrow represents the amplitude of the smallest mitoGCaMP6m transient (ΔF/*F* = 44 %), which decayed biexponentially. (**d**) The amplitude of spike-evoked mitoGCaMP6m fluorescence transients varies between different neuronal processes. *Left*, Localization of the mitoGCaMP6m labeled mitochondria in a representative L5 pyramidal neuron. The rectangles indicate the regions within the apical dendrite (cyan), basal dendrite (magenta), and axon initial segment (red) from which fluorescence measurements were obtained. *Right*, Mitochondrial and cytosolic (green) Ca^2+^ transients elicited by a train of 5 APs at 50 Hz in different neuronal compartments. (**e**) Mean peak ΔF/F of the mitoGCaMP6m transients as a function of the number of action potentials in soma, apical, and basal dendrites, and axon initial segment. Shown are mean values ± SE (n = 5–29).

To determine how neuronal electrical activity affects mitochondrial Ca^2+^ dynamics, we performed somatic whole-cell recordings from L5 pyramidal neurons expressing the mitochondria-targeted, neuron-specific Ca^2+^ indicator, mitoGCaMP6m. The intracellular solution was supplemented with the cytosolic Ca^2+^ indicator, Fura-2, that could be excited separately from mitoGCaMP6m. Series of thin, high-resolution optical sections over the vertical extent of the neuron, representing Fura-2 and mitoGCaMP6m fluorescence elicited by two-photon excitation at 760 and 960 nm, respectively, were used to reconstruct the morphology of the neuron and to reveal the position of the individual mitochondria within its soma and processes. To improve the temporal resolution of the optical signals, dynamic fluorescence measurements were obtained from the smaller regions of interest in soma, axon, and dendrites. In a typical experiment, two APs elicited by the injection of two brief current pulses via the patch pipette caused cytosolic and mitochondrial Ca^2+^ elevations in the soma of an L5 cell (Figure 1a). Although the cytosolic Ca^2+^ signals had relatively even intensity, the change in mitoGCaMP6m fluorescence occurred at ‘hotspots’, each representing an individual mitochondrion. The mitochondrial Ca^2+^ elevations began with a short delay after the beginning of the spike train, and they were observed only in the electrically active neurons. At the same time, the fluorescence of the nearby mitoGCaMP6m expressing mitochondria belonging to the non-active cells did not change (Figure 1—figure supplement 1).

**Figure 1—figure supplement 1.**
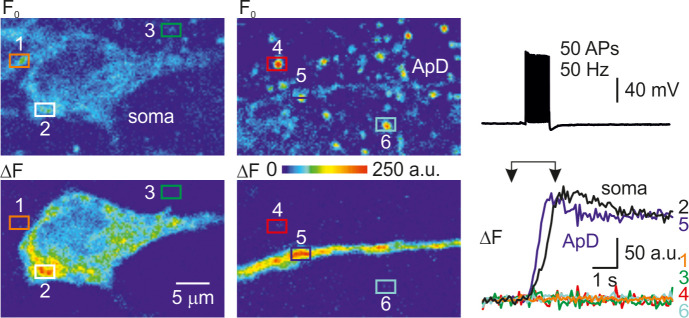
Ca^2+^ elevations in the mitochondria of the electrically active neurons. *Left*, Pseudocolor maps of the mitoGCaMP6m resting fluorescence (Fo, top) and of the change in fluorescence elicited by a train of 50 APs at 50 Hz (ΔF, bottom) between the times marked by the arrowheads in the right panel. The rectangles 1–6 indicate the regions of interest from which fluorescence measurements were obtained. ROIs 2 and 5 contain a single mitochondrion within the soma and apical dendrite, respectively, of the electrically active, patched neuron. ROIs 1,3,4,6 contain mitochondria belonging to the nearby, electrically inactive cells. *Right*, Action potentials elicited by a train of current pulses injected via the somatic whole-cell pipette only trigger Ca^2+^ elevation in the mitochondria of the electrically active neuron (ROI 2, 5).

**Figure 1—figure supplement 2.**
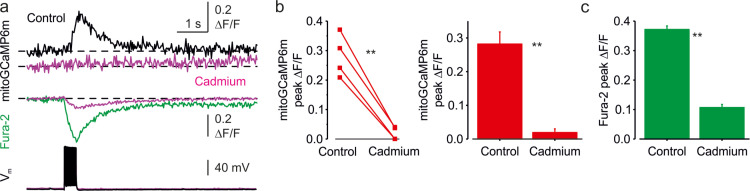
Blockade of voltage-gated Ca^2+^ channels abolishes spike-evoked mitochondrial Ca^2+^ transients. (**a**) Somatic mitoGCaMP6m (*top*) and Fura-2 (*bottom*) transients elicited by a train of 20 APs before (black and green respectively) and, 20 minutes after (magenta) bath application of Cadmium (200 µM). (**b**) Cadmium application blocks mitochondrial Ca^2+^ transients. *Left,* Peak amplitude mitoGCaMP6m ΔF/F transients elicited by 20 AP before and after application of Cadmium. Notice that the mitochondrial Ca^2+^ transients were almost completely abolished when Cadmium is present. A line connects the paired values obtained from the same individual neuron. Right, Mean peak amplitude of mitoGCaMP6m ΔF/F transients in control*,* after the Cd^2+^ application (28% ± 4% vs 2 ± 1%, mean ± SE, n = 4, p < 0.01). (**c**) Cadmium application blocks cytosolic Ca^2+^ transients. Mean peak amplitude of Fura-2 ΔF/F transients in control*,* and after the Cd^2+^ application was significantly suppressed (37% ± 1% vs 11 ± 1%, mean ± SE, n = 3, p < 0.01).

**Figure 1—figure supplement 3.**
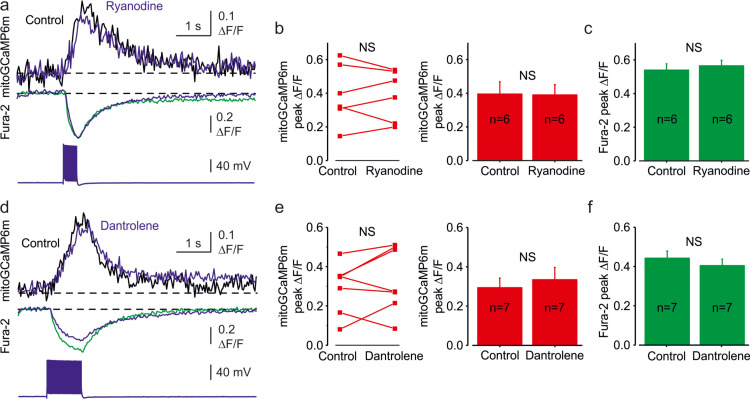
Blockade of the ER Ryanodine receptors has no significant effect on mitochondrial Ca^2+^ transients. (**a**) Somatic mitoGCaMP6m transients elicited by a train of 20 APs before (black) and 20 min after (blue) bath application of high concentration of Ryanodine (100 µM). (**b**) Blockade of Ryanodine receptor-mediated calcium release from the endoplasmic reticulum did not affect mitochondrial Ca^2+^ transients. *Left,* The peak amplitude of mitoGCaMP6m ΔF/F transients before and after application of Ryanodine. A line connects the paired values obtained from the same individual neuron. *Right*, Mean peak amplitude of mitoGCaMP6m ΔF/F transients in control (39% ± 7%, n = 6) and after the Ryanodine application (39% ± 6%, n = 6, p = 0.88). (**c**) Mean peak amplitude of Fura-2 ΔF/F transients in control and after the Ryanodine application (53% ± 4% vs 57 ± 3%, respectively, n = 6, p = 0.37). Notice that Ryanodine produces no significant change in the amplitude of the cytosolic Ca^2+^ transients. (**d**) Somatic mitoGCaMP6m transients elicited by a train of 50APs before (black) and 20 min after (blue) bath application of Ryanodine receptor blocker, Dantrolene (100 µM). (**e**) Blockade of Ryanodine receptor-mediated calcium release from the endoplasmic reticulum by Dantrolene did not affect mitochondrial Ca^2+^ transients. *Left,* The peak amplitude of mitoGCaMP6m ΔF/F transients before and after application of Dantrolene. A line connects the paired values obtained from the same individual neuron. *Right*, Mean peak amplitude of mitoGCaMP6m ΔF/F transients in control (29% ± 5%, n = 7) and after the Dantrolene application (33% ± 6%, n = 7, p = 0.33). (**f**) Mean peak amplitude of Fura-2 ΔF/F transients in control and after the Dantrolene application (44% ± 4% vs 40 ± 3%, respectively, n = 7, p = 0.16). Notice that Dantrolene produces no significant change in the amplitude of the cytosolic Ca^2+^ transients.

**Figure 1—figure supplement 4.**
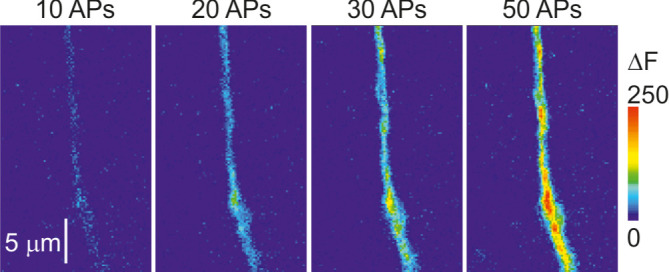
The amplitude of mitoGCaMP6m transients varies as a function of the number of APs. Shown are pseudocolor maps of change in the mitoGCaMP6m fluorescence in response to trains of 10, 20, 30, and 50 APs at 50 Hz. Fluorescence was measured in the same apical dendrite as in Figure 1—figure supplement 1. Notice that the amplitude of the ΔF transients increases as a function of the number of spikes in the train.

**Figure 1—figure supplement 5.**
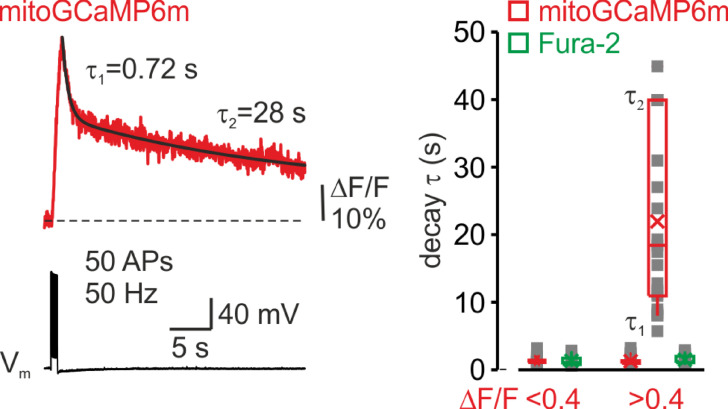
The decay time course of mitochondrial Ca^2+^ transients varies as a function of their amplitude. *Left*, the double exponential time course of mitoGCaMP6m transient elicited by 50 APs during a prolonged (35 s) optical recording. *Right,* The decay time course of the mitoGCaMP6m and Fura-2 transients. Each dot (gray) represents a decay time constant (τ for monoexponential decays, τ _1_ and τ _2_ for bi-exponential decays) of transients elicited by 5–50 APs in 42 neurons. Note that the smaller mitoGCaMP6m transients (peak ΔF/*F* < 0.4, n = 25) decayed mono-exponentially, whereas the decay of the larger mitoGCaMP6m transients (peak ΔF/*F* > 0.4, n = 22) followed the bi-exponential time course. The decay of Fura-2 transients was rapid and followed the monoexponential time course, independently of their amplitude. Box plots represent the 25–75% interquartile range of the τ, τ_1_, and τ_2_ values for mitoGCaMP6m (red) and Fura-2 (green) transients, and the whiskers expand to the 5–95% range. A horizontal line inside the box represents the median of the distribution, and the mean is represented by a cross symbol (X).

**Figure 1—figure supplement 6.**
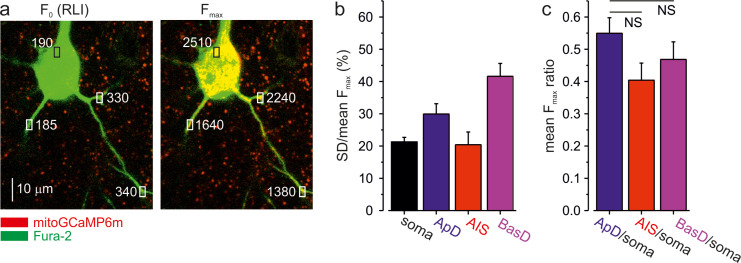
MitoGCaMP6m is homogenously expressed in soma and processes of L5 pyramidal neurons. (**a**) Individual mitoGCaMP6m-labeled mitochondria in a representative voltage-clamped L5 pyramidal neuron at a holding potential of –70 mV and following a one-minute long voltage step +20 mV. The images were obtained by merging the single, 1-µm-thick optical sections at excitation wavelengths of 760 and 960 nm, eliciting the Fura-2 (green) and mitoGCaMP6m (red) fluorescence, respectively. An increase in yellow color intensity following the prolonged voltage step reflects an increased mitoGCaMP6m fluorescence. Rectangles indicate the regions within the thin basal dendrite and somata, from which fluorescence measurements (white numbers) were obtained. Notice that the prolonged depolarization elicited a 4–12 times increase in mitoGCaMP6m fluorescence in all neuronal compartments. (**b**) Variance analysis of the maximal mitoGCaMP6m fluorescence (F_max_) reveals little difference in mitoGCaMP6m expression in the individual mitochondria in the soma and neuronal processes. F_max_ values were measured from 5 to 70 individual mitochondria in soma, apical and basal dendrites and the AIS of 14 neurons following prolonged depolarizing voltage step. The mean SD/mean of F_max_ was 21% ± 1% for somatic mitochondria, 30% ± 3% in apical dendrites, 20% ± 4% in AIS, and 42% ± 4% in basal dendrites. The relatively narrow F_max_ distributions reflect homogeneity in mitoGCaMP6m expression within the mitochondrial population. (**c**) Mean ratio of F_max_ in the neuronal process vs. soma. The mean F_max_ ratio was 0.55 ± 0.05 for mitochondria in apical dendrites vs. soma, 0.40 ± 0.05 for mitochondria in AIS vs. soma, 0.47 ± 0.05 for mitochondria in basal dendrites vs. soma (mean ± SE, n = 14 neurons).

The AP-elicited mitochondrial Ca^2+^ elevations required Ca^2+^ influx from the extracellular space. It is likely that Ca^2+^ ions enter via the voltage-gated Ca^2+^ channels, as bath application of Cd^2+^ (200 µM) inhibited both cytosolic and mitochondrial Ca^2+^ transients (Figure 1—figure supplement 2). Previous studies have shown that Ca^2+^ depletion of the endoplasmic reticulum (ER) in cultured neurons does not influence mitochondrial Ca^2+^ elevations (Ashrafi et al., 2020). Consistent with these results, we found that blockade of ER Ca^2+^ channels by Ryanodine (100 µM) and Dantrolene (100 µM) (Figure 1—figure supplement 3) had no significant effect on mitochondrial Ca^2+^ transients.

An increase in the number of spikes produced progressively larger mitoGCaMP6m transients in the soma (Figure 1b) and the apical dendrite (Figure 1—figure supplement 4) with a progressively slower rising phase. Thus, the mito-Ca^2+^ signals elicited by 50 APs grew throughout the spike train duration, reaching a peak ΔF/F value of 59% ± 11% (n = 29) and 54% ± 11% (n = 22) for soma and apical dendrite, respectively, at 120 ± 18ms (n = 10) after the train end. In contrast with previous reports (Ashrafi et al., 2020; Lin et al., 2019), mito-Ca^2+^ transients elicited by 2–20 spikes decayed as rapidly or even faster than cytosolic Ca^2+^ transients. However, following large elevations, [Ca^2+^]_m_ remained high for tens of seconds, reaching the resting level long after the cytosolic Ca^2+^ concentration completely recovered. We systematically examined the relationship between the peak amplitude and the decay rate of the mito-Ca^2+^ transients in 42 pyramidal neurons (Figure 1c, Figure 1—figure supplement 5). Decay of smaller transients (peak ΔF/*F* < 40%) was always monoexponential, with *τ* = 0.86 ± 0.11 s (n = 25) as well as decay of some middle-sized (ΔF/F 40–75%) transients (*τ* = 0.82 ± 0.10 s, n = 10). The decay of other middle size and large (ΔF/*F* > 75%) transients was double exponential, with τ_1_ = 0.86 ± 0.13 s and τ_2_ = 22.0 ± 2.85 s (n = 23). The decay of cytosolic Ca^2+^ transients always followed a single exponential time course which was characterized by *τ* = 1.03 ± 0.14 s (n = 22) for small and *τ* = 1.19 ± 0.4 s (n = 20, p = 0.43) for large transients.

To elucidate the differences in mitochondrial Ca^2+^ signaling between distinct neuronal compartments, we monitored mitoGCaMP6m fluorescence in ~10 µm long regions of interest in basal, apical dendrites, and axon initial segments (AISs) during trains of five APs (Figure 1d). While the amplitude and time course of the cytosolic Ca^2+^ responses in all these compartments were similar, the magnitude of mitochondrial signals was remarkably polar. The amplitude of the mitochondrial Ca^2+^ elevations in apical dendrite was as prominent as in the soma. In contrast, in the AIS and thin basal dendrites, the mito-Ca^2+^ responses were dramatically smaller.

We next sought to evaluate the relationship between the number of APs and the mean peak amplitude of the mitoGCaMP6m transients in the soma, AIS, apical and basal dendrites of 29 neurons (Figure 1e). The steepness of this relationship was greater in soma and apical dendrites (1.3% and 1.1% ΔF/F per spike, respectively) compared with AIS and basal dendrites (0.4% and 0.3% ΔF/F per spike, respectively). The compartmental differences in mitochondrial signals were not due to a different magnitude of cytosolic Ca^2+^ elevations. The pattern of [Ca^2+^]_i_ during the neuronal activity is known to be complex. However, the differences in peak cytosolic Ca^2+^ levels were subtle, and they poorly correlated with the magnitude of mitochondrial Ca^2+^ elevations. For example, the peak ΔF/F amplitude of Fura-2 transients elicited by twenty spikes was 26% ± 3.6% (n = 22) for soma, 37% ± 4% (n = 13) for apical dendrite, 38% ± 4% (n = 13) for basal dendrites, and 25% ± 4% in the AIS (n = 7). The compartmental differences in magnitude of the mitoGCaMP6m transients could, at least partially, be explained by the lower expression level of fluorescence probe in the mitochondria localized within the thinner neuronal processes. This seems to be unlikely, however, since, in all neuronal compartments, maximal mitoGCaMP6m fluorescence of the individual mitochondria following prolonged depolarization of the neuronal membrane was similar (Figure 1—figure supplement 6). Our evidence, therefore, points to the existence of an as-yet-unidentified mechanism that differentially regulates the mitochondrial Ca^2+^ entry in distinct neuronal compartments.

### Frequency-dependent amplification of mitochondrial Ca^2+^ elevations

**Figure 2.**
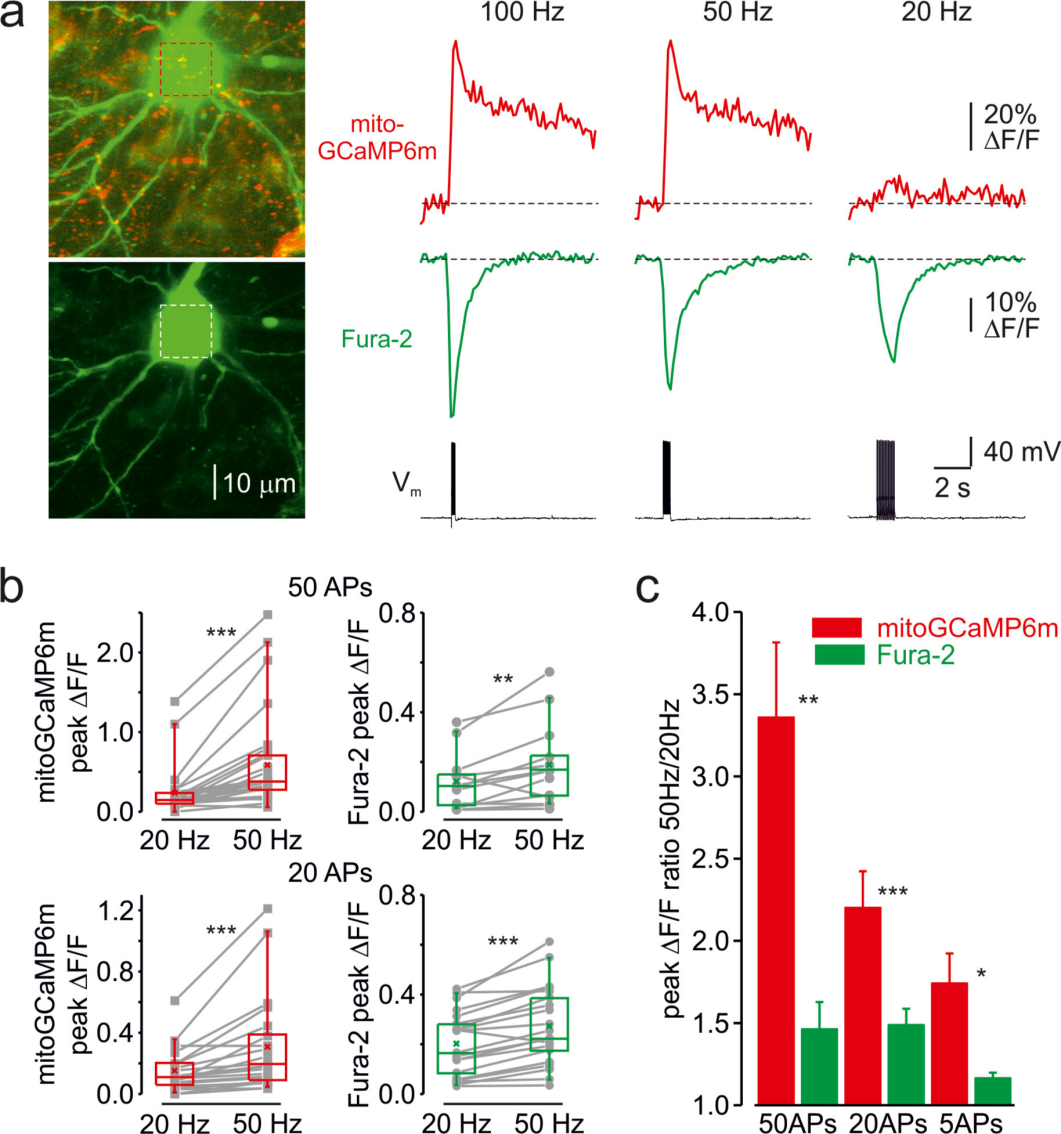
Frequency-dependent amplification of the mitochondrial Ca^2+^ elevations. (**a**) Cytosolic and mitochondrial Ca^2+^ elevations elicited at the soma of a representative pyramidal neuron by trains of 20 APs at 100, 50, and 20 Hz. *Left*, The top image is mitoGCaMP6m labeled mitochondrial map of a representative L5 pyramidal neuron, obtained by merging the Fura-2 and mitoGCaMP6m fluorescence (for detail, see Figure 1). The bottom image is the maximum intensity Z-projection representing the Fura-2 fluorescence only. *Right*, The mitoGCaMP6m (red) and Fura-2 (green) ΔF/F transients elicited at the soma by the spike trains at indicated frequency. Notice that, at 20 Hz, the train of spikes produced almost no mitochondrial Ca^2+^ elevation, while the amplitude of the cytosolic Ca^2+^ transient changed little as a function of spike frequency. (**b**) Peak amplitude of mitoGCaMP6m (red) and Fura-2 (green) ΔF/F transients elicited by a train of 50 (top) or 20 (bottom) APs at a frequency of 20 and 50 Hz. The gray lines connect the paired values obtained from the same individual neuron at two firing frequencies. Box plots represent the 25–75% interquartile range, and the whiskers expand to the 5–95% range. A horizontal line inside the box represents the median of the distribution, and the mean is represented by a cross symbol (X). (**c**) Mean ratio of peak amplitudes of mitoGCaMP6m (red) and Fura-2 (green) ΔF/F transients elicited by trains of 50, 20, and 5 APs at 50 and 20 Hz frequency.

Next, we examined whether mitochondrial Ca^2+^ elevations are sensitive to firing frequency. Figure 2a shows an optical recording from the soma of a representative L5 neuron in which cytosolic and mitochondrial Ca^2+^ transients were elicited by trains of twenty APs at 20, 50, and 100 Hz. As spike frequency increased, the rise of cytosolic Ca^2+^ transients became progressively faster and their peak amplitude modestly increased (Figure 2—figure supplement 1). In contrast, the mitochondrial Ca^2+^ signals showed a very different frequency dependence. Firing at a frequency of 20 Hz or lower elicited a minimal elevation in [Ca^2+^]_m_, whereas the response to spikes at a frequency of 50 Hz or higher was dramatically greater. The steep frequency dependence of the mitochondrial signals was observed in all neuronal compartments, including somas, apical, and basal dendrites, making it unlikely that it reflects the frequency-dependent failure of AP backpropagation (Spruston et al., 1995). The frequency-dependent amplification of mitochondrial Ca^2+^ transients was observed in all 21 neurons tested with either 50, 20, or 5 APs (Figure 2b). The mean ratio of peak amplitudes of the mitochondrial Ca^2+^ transients elicited by trains of spikes at 50 and 20 Hz was larger when neurons were subjected to longer (3.36 ± 0.46 times, n = 22 for 50 APs) than to shorter (1.74 ± 0.18 times, n = 5 for 5 APs) trains (Figure 2c). Systematic varying of AP frequency in a range from 10 to 100 Hz revealed that the peak amplitude of mito-Ca^2+^ transients behaved as Bolzmannian function of the frequency, with mean half-amplitude of 38 Hz and steepness of 22 Hz^–1^ (n = 12).

**Figure 2—figure supplement 1.**
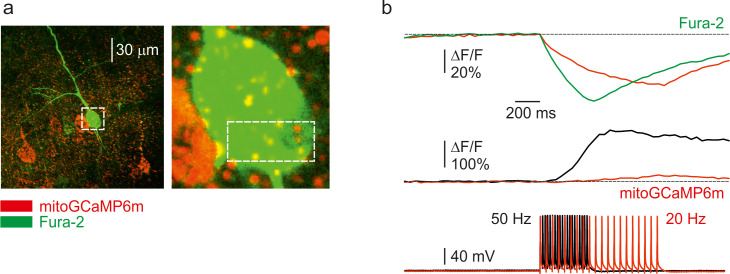
The amplitude of the mitochondrial Ca^2+^ elevation is proportional to the rate-of-rise of the cytosolic Ca^2+^ concentration. (**a**)*Left,* an image of a representative L5 pyramidal neuron, obtained by merging the Fura-2 and mitoGCaMP6m fluorescence (for detail, see Figure 1). *Right,* inset: higher magnification image, corresponding to the rectangle in the left panel. The rectangle indicates the region from which the fluorescence measurements were obtained. (**b**) Δ*F*/*F* transients elicited by a train of 20AP at 20 Hz (red) and 50 Hz (black and green). Cytosolic (top) and mitochondrial (bottom) Ca^2+^ transients. Notice the slower time course of the cytosolic calcium transient in response to a 20 Hz spike train. The slower rise, and not the difference in [Ca^2+^]_i_ level, correlates with the mitochondrial calcium transient amplitude.

**Figure 2—figure supplement 2.**
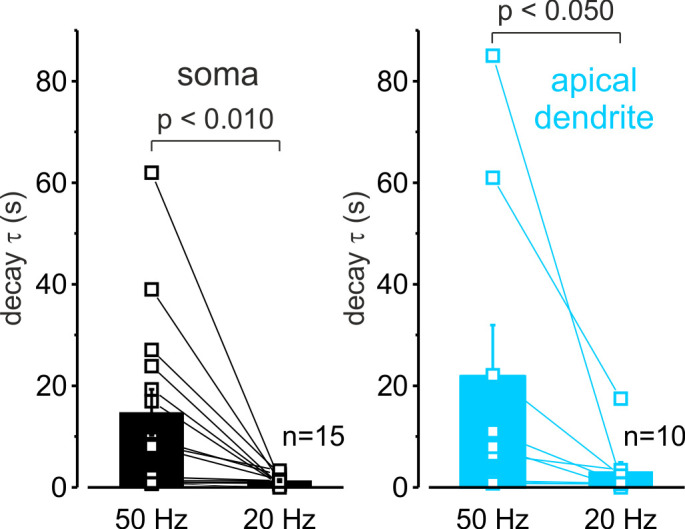
High-frequency firing elicits slowly decaying mitochondrial Ca^2+^ transients in soma and dendrites of L5 pyramidal neurons. The decay time constant of somatic (left, black) and dendritic (right, cyan) mitoGCaMP6m transients elicited by trains of 50 APs at a frequency of 20 and 50 Hz. The lines connect paired values obtained from the same neuron at two firing frequencies. The bars represent the mean values ± SEM.

The decay time course of the mitochondrial Ca^2+^ transients elicited by high-frequency spike trains was significantly slower than those produced by the same number of spikes at a lower frequency (Figure 2—figure supplement 2). Thus, trains of fifty APs at 50 Hz elicited the extremely slowly decaying mito-Ca^2+^ transients (*τ* > 10 s) in 11/15 somas and 7/10 apical dendrites. In contrast, all but one mito-Ca^2+^ transient produced in the same neurons by fifty APs at 20 Hz decayed rapidly.

### Frequency-dependent acceleration of the mitochondrial NAD(P)H metabolism

**Figure 3.**
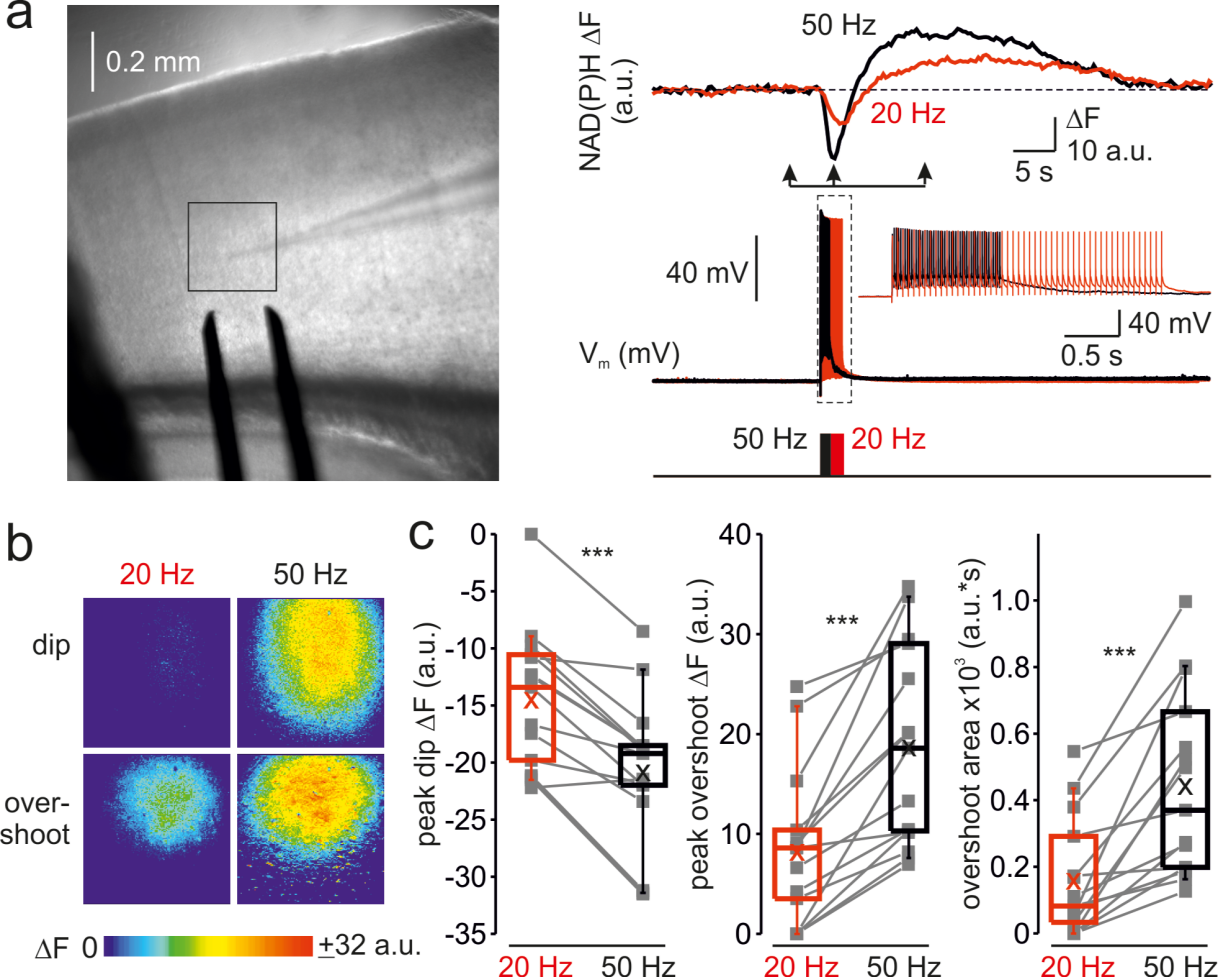
Frequency-dependent amplification of spike elicited changes in mitochondrial NAD(P)H auto-fluorescence. (**a**) In a representative cortical slice, changes in NAD(P)H fluorescence in response to extracellular stimuli trains depend on stimulation frequency. *Left,* DIC image of a coronal slice during the electrical and optical recording. The rectangle indicates the region from which the auto-fluorescence measurements were obtained. The stimuli were delivered via the bipolar electrode placed on the white-gray matter border, and the whole-cell recording was obtained from an L5 neuron within the same cortical column. *Right*, The membrane potential and optical traces evoked by trains of 50 just suprathreshold stimuli at 50 Hz (black) and 20 Hz (red). Notice that both dip and overshoot of the NAD(P)H signals are more prominent at 50 Hz. *Inset*: Stimuli intensity was carefully adjusted to elicit only a single AP per stimulus. (**b**) The amplitude of NAD(P)H signals depends on stimulation frequency, whereas their spatial extent does not. Shown are pseudocolor maps of change in the NAD(P)H fluorescence between the times marked by the arrowheads in **a**. (**c**) Higher frequency stimulation causes an increase in the magnitude of the dip and of the overshoot of the NAD(P)H signal. Box plots representing the peaks of the dip (left), the peaks of the overshoot (middle), and the area of the overshoot (right) of the NAD(P)H signals evoked by trains of 20 Hz (red) and 50 Hz (black) stimuli. The gray lines connect the paired values obtained from the same cortical regions at two firing frequencies (n = 15 ROIs, ten cortical slices, three mice). Box plots represent the 25–75% interquartile range, and the whiskers expand to the 5–95% range. A horizontal line inside the box represents the median of the distribution, and the mean is represented by a cross symbol (X).

To determine whether the frequency-dependent amplification of Ca^2+^ signals in the mitochondria affects their metabolic activity, we monitored the changes in NAD(P)H autofluorescence elicited by trains of brief, just suprathreshold antidromic stimuli at a different frequency (Figure 3a). Whole-cell recording from a single pyramidal neuron within the region of interest was obtained to tune the stimuli intensity such that each stimulus would elicit only one AP. In cortical neurons, NAD(P)H signals primarily reflect changes in mitochondrial NAD(P)H pool (Díaz-García et al., 2021). In response to electrical stimulation, we observed a negative deflection in the NAD(P)H autofluorescence (‘dip’) which indicates an increased rate of electron transfer reflected in NAD(P)H oxidation, followed by a positive transient (‘overshoot’) which indicates Krebs-cycle-dependent replenishment of NAD(P)H pool. At higher stimulation frequency, the magnitude of both dip and overshoot of the NAD(P)H signals were enhanced, consistent with the previous reports that the rates of NAD(P)H oxidation and synthesis are dependent on the Ca^2+^ level in the mitochondrial matrix (Díaz-García et al., 2021). Spatio-temporal analysis of the NAD(P)H autofluorescence dynamics at two stimulation frequencies (Figure 3b) revealed that the changes in the fluorescence were spatially restricted to Layer 5 of a single cortical column and that the frequency-dependent amplification of both dip and overshoot amplitude was relatively uniform within the stimulated region. A comparison of NAD(P)H autofluorescence changes in response to a train of 50 stimuli at 20 Hz and 50 Hz in ten cortical slices (Figure 3c) revealed the same frequency dependence as with [Ca^2+^]_m_. The dip amplitude increased from –15 ± 2 a.u. at 20 Hz to –21 ± 2 a.u. at 50 Hz (n = 15 ROIs, p < 0.001), the overshoot’s peak amplitude increased from 8 ± 2 a.u. to 19 ± 3 a.u. (p < 0.001) and the overshoot area increased from 156 ± 46 a.u.∙s to 441 ± 71 a.u.∙s (p < 0.001), respectively. Because the glial responses might partially contaminate NAD(P)H signals obtained in the cortical slices, we tested the frequency dependence of NAD(P)H responses in the in Stratum pyramidale of CA1 area of the hippocampus (Figure 3—figure supplement 1) which predominantly contains neuronal cell bodies. As in the neocortex, NAD(P)H signals in response to trains of stimuli delivered to the Stratum oriens were significantly enhanced at the higher stimulation frequency.

**Figure 3—figure supplement 1.**
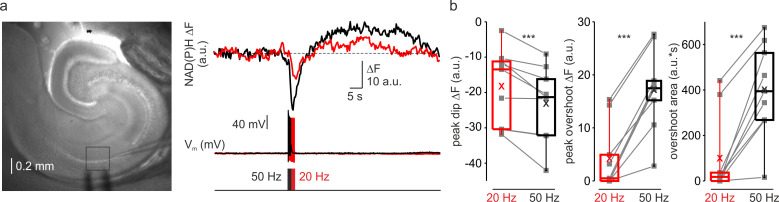
Firing frequency dependent amplification of NAD(P)H signal in the CA1 area of hippocampus. (**a**) In a representative hippocampal slice, changes in NAD(P)H fluorescence in response to extracellular stimuli trains depend on stimulation frequency. *Left,* DIC image of a hippocampal slice during the electrical and optical recording. The rectangle indicates the region from which the auto-fluorescence measurements were obtained. The stimuli were delivered via the bipolar electrode placed in Stratum oriens, and the whole-cell recording was obtained from a nearby CA1 pyramidal neuron. *Right*, The membrane potential and optical traces evoked by trains of 50 just suprathreshold stimuli at 50 Hz (black) and 20 Hz (red). Notice that both dip and overshoot of the NAD(P)H signals are more prominent at 50 Hz. (**b**) Higher frequency stimulation causes an increase in the magnitude of the dip and of the overshoot of the NAD(P)H signal. Box plots representing the peaks of the dip (left, –18 ± 4 and –23 ± 4 a.u. for 20 and 50 Hz, respectively, n = 9, p < 0.005), the peaks of the overshoot (middle, 4 ± 2 and 17 ± 3 a.u. for 20 and 50 Hz, respectively, n = 9, p < 0.001), and the area of the overshoot (right, 100 ± 59 and 401 ± 69 a.u.*s for 20 and 50 Hz, respectively, n = 9, p < 0.001) of the NAD(P)H signals. The gray lines connect the paired values obtained from the same regions at two firing frequencies. Box plots represent the 25–75% interquartile range, and the whiskers expand to the 5–95% range. A horizontal line inside the box represents the median of the distribution, and the mean is represented by a cross symbol (X).

### Localized dendritic [Ca^2+^]_m_ elevations elicited by the coincidence of postsynaptic AP and EPSP

**Figure 4.**
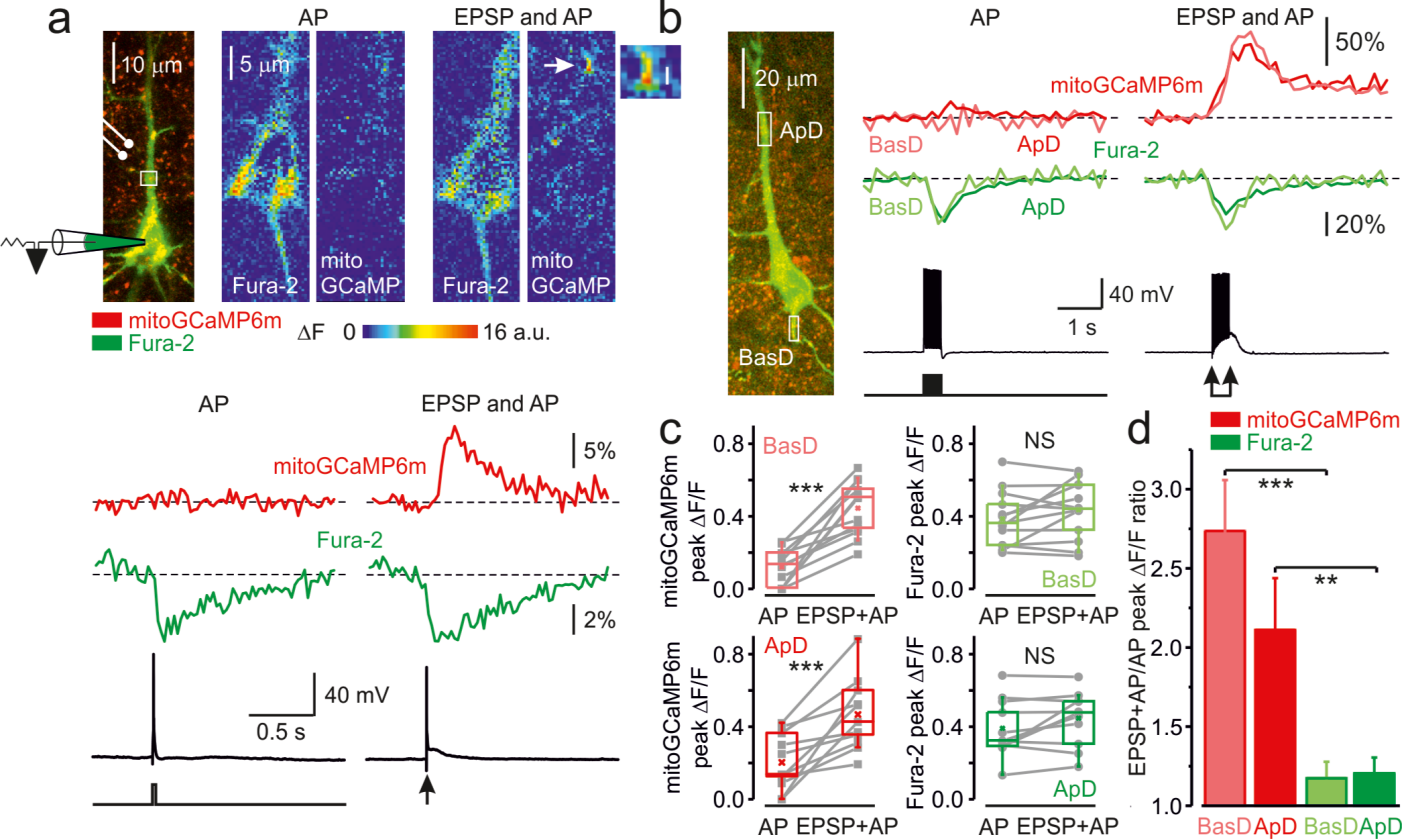
The coincidence of postsynaptic action potential and EPSP induces localized mitochondrial Ca^2+^ elevations in the dendrites. (**a**) Action potential elicited by a single, just suprathreshold synaptic stimulus induces a large, spatially restricted increase in dendritic mitoGCaMP6m fluorescence, whereas action potential evoked by a 5 ms current pulse (600 pA, cell body injection) had no such effect. *Top, Left*: Mitochondrial map in a representative L5 pyramidal neuron, obtained by merging the Fura-2 and mitoGCaMP6m fluorescence (for detail, see Figure 1). The rectangle indicates the region within the apical dendrite from which fluorescence measurements were obtained. *Right:* Pseudocolor maps of change in the mitoGCaMP6m and Fura-2 fluorescence in response to a synaptically and current pulse evoked AP. *Bottom*, The mitoGCaMP6m (red) and Fura-2 (green) ΔF/F transients measured from regions of interest as indicated in the upper panel and the somatic membrane potential trace. Arrow indicates a hotspot at which the synaptic stimulus elicited a mitochondrial Ca^2+^ transient. The transients are ensemble averages of 50 sweeps. (**b**) Action potentials elicited by a train of synaptic stimuli produce larger mitoGCaMP6m signals in the dendrites than APs elicited by the injection of a train of current pulses. *Left*: Mitochondria map in a representative L5 pyramidal neuron, obtained by merging the Fura-2 and mitoGCaMP6m fluorescence. The rectangles indicate the regions within the apical and basal dendrites from which fluorescence measurements were obtained. *Right*, Comparison of ΔF/F mitoGCaMP6m transients elicited in the apical (red) and basal dendrite (rose) by twenty suprathreshold synaptic stimuli at 50 Hz and by twenty brief current pulses delivered to the soma. The green and light green traces are ΔF/F Fura-2 transients in apical and basal dendrites, respectively. The black traces represent somatic membrane potential. (**c**) Peak amplitude of mitoGCaMP6m and Fura-2 ΔF/F transients elicited in basal (top) and apical dendrites (bottom) by a train of twenty suprathreshold current pulses or synaptic stimuli at a frequency of 50 Hz. The line connects the paired values obtained from the same individual neuron at two stimulation modalities. Box plots represent the 25–75% interquartile range, and the whiskers expand to the 5–95% range. A horizontal line inside the box represents the median of the distribution, and the mean is represented by a cross symbol (X). (**d**) Mean ratio of peak amplitudes of mitoGCaMP6m (rose, red) and Fura-2 (light green, green) ΔF/F transients elicited in dendrites by suprathreshold synaptic stimuli (EPSP + AP) and current pulses (AP). Data obtained from 8 to 13 individual neurons.

We next sought to elucidate how synaptic activity affects mitochondrial Ca^2+^ dynamics. After filling the cell for ~20 min to allow the diffusion of Fura-2 into the dendrites, we positioned the bipolar electrode close to an apical or basal dendrite. Delivery of a single brief stimulus (0.1ms), with its amplitude adjusted to keep the subsequent EPSP below the threshold for postsynaptic spike generation produced no detectable cytosolic or mitochondrial Ca^2+^ signals (n = 7). The single AP elicited by brief somatic current pulse injection, however, produced a cytosolic but no mitochondrial Ca^2+^ response in the dendrites. Remarkably, the coincidence of the EPSP and backpropagating AP synergized to elicit a robust cytosolic and mitochondrial Ca^2+^ response (Figure 4a). While most dendritic mitochondria were silent, the EPSP and AP coincidence created single localized mitochondrial Ca^2+^ “hotspots” with peak ΔF/F amplitude of 6.4% ± 0.8% (n = 8) in the dendritic regions of interest. We interpreted the appearance of these hotspots as evidence for a highly restricted, probably single mitochondrial Ca^2+^ elevation, in the vicinity of the active spine. The unitary character of the mitochondrial signals under this experimental paradigm can be explained by a spatial sparseness of the synapses formed by the presynaptic fiber on the dendrites of the postsynaptic cortical cell (Markram et al., 1997a) such that only one out of a few currently active spines could be found in the relatively short segment of a dendritic branch that we examined. The cytosolic Ca^2+^ elevation, measured in the same hotspot at which the mitoGCaMP6m signal was detected, was not significantly larger than in the nearby dendrite. The failure to see the cytosolic Ca^2+^ “hotspots” is, most probably, due to temporal and amplitude resolution of our optical recording that was insufficient to reveal Ca^2+^ elevation in the tiny volume between the spine neck and the mitochondrion during the fast single spine Ca^2+^ transient (Miyazaki and Ross, 2017; Svoboda et al., 1996). We extended our analysis by measuring the cytosolic and mitochondrial Ca^2+^ signals elicited by 20 unpaired APs and APs paired with EPSP. In both apical and basal dendrites, the paired APs produced a significantly larger mitochondrial signal. In contrast, the cytosolic Ca^2+^ elevation amplitude was not significantly different for unpaired and paired stimulation (Figure 4b and c). Hence, the peak amplitude of the mitoGCaMP6m transients elicited by paired APs was 2.73 ± 0.32 (n = 8) and 2.11 ± 0.32 (n = 9) times higher than of those evoked by the unpaired APs in the basal and apical dendrites, respectively (Figure 4d). The ability of postsynaptic neurons to generate an AP was crucial for triggering the dendritic mito-Ca^2+^ transients. Thus, intracellular dialysis with a solution containing a blocker of voltage-gated Na^+^ channels, QX-314, which prevented the firing of the postsynaptic cells (Connors and Prince, 1982) while producing only a minor effect on the EPSP generation, dramatically reduced the amplitude of synaptically evoked mito-Ca^2+^ transients (Figure 4—figure supplement 1). As with single subthreshold EPSPs, even the significantly larger EPSPs evoked in QX-314 dialyzed neurons by a train of strong synaptic stimuli, in the absence of AP, produced no significant Ca^2+^ elevation in dendritic mitochondria.

**Figure 4—figure supplement 1.**
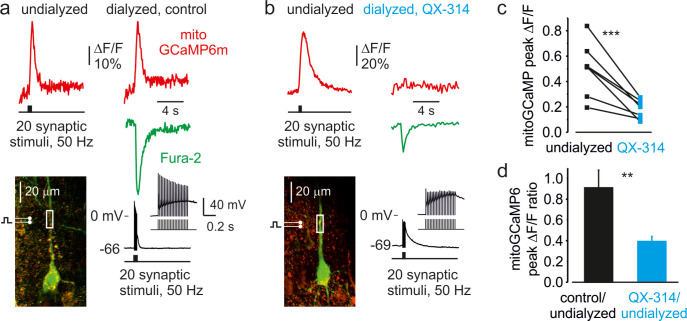
EPSPs alone cause no significant Ca^2+^ elevation in dendritic mitochondria. (**a**) MitoGCaMP6m Δ*F*/*F* transients (red) in the apical dendrite elicited by a train of 20 synaptic stimuli before (*left*) and 10 min after (*right*) break-in to the whole-cell configuration with a pipette filled with control solution. Note that the mitochondrial Ca^2+^ transients are almost unaffected by intracellular dialysis. The green trace represents the Fura-2 Δ*F*/*F* transient obtained after the establishment of whole-cell recording. *Inset*: Whole-cell voltage recording of a sequence of EPSPs and orthodromic APs elicited by the train of stimuli. (**b**) MitoGCaMP6m *ΔF/F* transients (red) in the apical dendrite elicited by a train of 20 synaptic stimuli before (*left*) and 10 min after (*right*) break-in to the whole-cell configuration with a pipette filled with QX-314 (100 µM) containing solution. QX-314, a Na^+^ channel blocker, was added to block postsynaptic AP generation. Note that, in the presence of QX-314, a train of large-amplitude EPSPs (see *inset*) was sufficient to produce a cytosolic Ca^2+^ elevation (green trace). However, the amplitude of mitochondrial Ca^2+^ transients was significantly reduced. (**c**) The peak amplitude of the dendritic mitoGCaMP6m ΔF/F transients elicited by 20 synaptic stimuli before (black) and after (blue) intracellular application of QX-314. Notice a significant decrease in amplitude of mitochondrial Ca^2+^ signals when AP generation is blocked (from 50 ± 8%–18 ± 3%, n = 7, p < 0.005). A line connects the paired values obtained from the same individual neuron. (**d**) Mean ratio of peak amplitudes of synaptically elicited, dendritic mitoGCaMP6m ΔF/F transients after the dialysis with either control (black, n = 8) or QX-314-containing intracellular solution (blue, n = 7) to those before the establishment of whole-cell recording. Notice that while the dialysis with control solution elicits little change in the amplitude of mitochondrial Ca^2+^ signals, blockade of postsynaptic AP causes a significant decrease in the ratio (p < 0.005).

**Figure 4—figure supplement 2.**
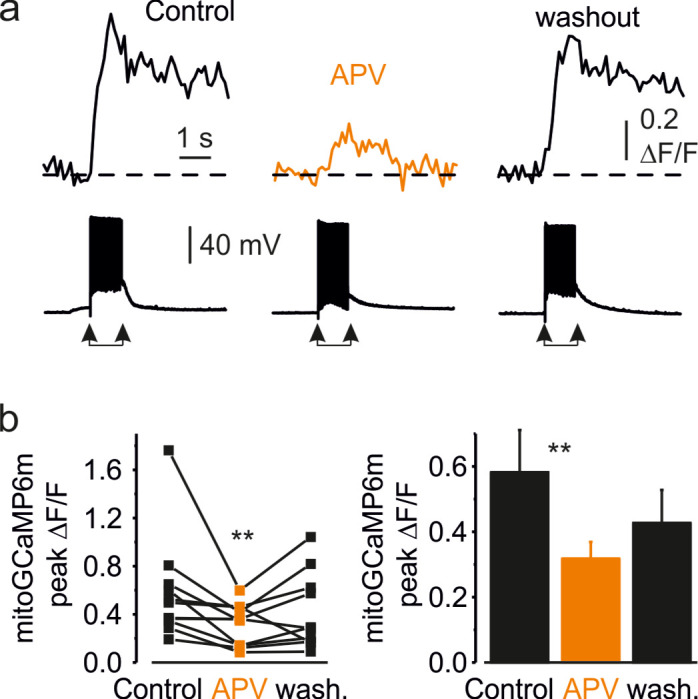
Availability of synaptic NMDA receptors is required for induction of dendritic mitochondrial Ca^2+^ elevation. (**a**) MitoGCaMP6m Δ*F*/*F* transients in the apical dendrite of a representative L5 neuron before, after bath application of APV (50 µM), and following the washout. The bottom traces represent somatic membrane potential. Arrows mark the beginning and end of the synaptic stimuli train. (**b**) Blockade of NMDA receptors causes a reduction in amplitude of the synaptically evoked mitochondrial Ca^2+^ transients in dendrites. *Left,* The peak amplitude of the dendritic mitoGCaMP6m ΔF/F transients elicited by 20 synaptic stimuli before, during APV application, and upon the washout. Notice a significant decrease in the transients' amplitude (n = 10, p < 0.01) when APV is present. A line connects the paired values obtained from the same individual neuron. *Right*, Mean peak amplitude of mitoGCaMP6m ΔF/F transients in control*,* after the APV application and after the washout (58% ± 13%, 32% ± 5% and 43% ± 10%, respectively, n = 10).

It is well established that the main route of Ca^2+^ entry into the spines is via the NMDARs (Mainen et al., 1999; Miyazaki and Ross, 2017), which require glutamate and depolarization to relieve the Mg^2+^ block (Nowak et al., 1984). Blockade of NMDARs by bath applied APV (50 µM) almost completely and reversibly abolished the mitoGCaMP6m transients evoked by the paired APs in the dendrites (Figure 4—figure supplement 2).

## Discussion

Using simultaneous electrical recordings, cytosolic and mitochondrial Ca^2+^ imaging in L5 pyramidal neurons, we found that relatively rare, singular spike firing, the firing mode associated with cortical circuit-based information processing in vivo (Brecht and Sakmann, 2002), produces fast-rising, rapidly decaying mitochondrial Ca^2+^ transients in all neuronal compartments. In contrast to a recent report of ‘loose’ coupling of mitochondrial Ca^2+^ transients to neuronal activity and cytosolic Ca^2+^ transients in in vivo cortical neurons (Lin et al., 2019), our data suggest a tight, causative relationship between neuronal electrical activity, cytosolic and mitochondrial Ca^2+^ levels.

Several studies (Ashrafi et al., 2020; Devaraju et al., 2017; Kwon et al., 2016; Lewis et al., 2018) have revealed the tens-of-seconds long mitochondrial Ca^2+^ transients in presynaptic terminals of cultured central neurons. While this extremely slow rate of Ca^2+^ clearance may reflect compartmental specialization of mitochondrial Ca^2+^ handling at the presynaptic sites, it seems more likely to be due to excessively intense stimulation. Indeed, small mitochondrial Ca^2+^ signals in axons of cultured hippocampal neurons elicited by 1–5 antidromic stimuli decayed rapidly (Gazit et al., 2016). Our results indicate that the prolonged mitochondrial Ca^2+^ elevations occur only following the long period of robust high-frequency firing. Although such intense firing is not typically observed in cortical pyramidal cells under physiological conditions, it may occur during various neurological diseases (Makinson et al., 2017; Zott et al., 2019) contributing to the mitochondrial Ca^2+^ overload and disease progression.

Our evidence indicates that the summation of the unitary AP-evoked mitochondrial Ca^2+^ transients steeply depends on firing frequency. Thus, in contrast to cytosolic Ca^2+^ signaling, which is less sensitive to firing rate, the mitochondrial Ca^2+^ elevations were strongly amplified at firing frequencies of >40 Hz. As expected, the enhanced mitochondrial Ca^2+^ uptake accelerated the rate of NAD(P)H production and consumption via the tricarboxylic acid cycle and the electron transport chain, respectively (Díaz-García et al., 2021), thereby upregulating the ATP synthesis.

The finding that action potential induced mitochondrial Ca^2+^ transients are about threefold greater in the soma and apical dendrites than in proximal axon and basal dendrites, most likely, indicates the compartmental difference in expression of mitochondrial Ca^2+^ channel, MCU. Alternatively, the subcellular differences in mitochondrial Ca^2+^ handling may reflect region-specific MCU molecular tuning (Ashrafi et al., 2020; Patron et al., 2019) or mitochondrial morphology (Lewis et al., 2018). From the functional viewpoint, the greater mitochondrial Ca^2+^ transients in thick neuronal processes might be necessary to compensate for the higher energetic cost of AP generation (Attwell and Laughlin, 2001).

A recent study suggests that the metabolic activity of mitochondria plays a pivotal role in synaptic plasticity (Rangaraju et al., 2019). How neuronal activity is linked to this process is poorly understood, however. Our results suggest that mitochondria can detect the Hebbian time coincidences between the pre- and postsynaptic spikes (Markram et al., 1997b). The resultant Ca^2+^ elevations in the mitochondrial matrix could be an essential part of the cascade of events underlying spike-time-dependent synaptic plasticity. This cascade might involve the initiation of fission of the dendritic mitochondria, as has been proposed for chemically induced NMDAR-dependent LTP in hippocampal neuronal culture (Divakaruni et al., 2018), although it remains unclear whether the LTP associated burst of fission events occurs at a physiologically relevant time scale. The involvement of the frequency-dependent mitochondrial Ca^2+^ signaling, most probably, explains the observed repetition rate requirement for LTP induction (Inglebert et al., 2020; Lisman and Spruston, 2005; Sjöström et al., 2001). Indeed, the spike-timing-dependent potentiation of cortical synapses, in addition to precise spike timing, requires a sufficiently high repetition frequency (Sjöström et al., 2001). Our data indicate that the unique ability of the mitochondria to decode firing frequency and Hebbian timing code of neuronal activity make this organelle a long-thought link between the firing pattern, metabolism, and plasticity.

## Materials and methods

### Experimental animals

All experiments were approved by the Animal Care and Use Committee of Ben Gurion University of the Negev. C57BL/6 mice were obtained from Envigo (Israel).

### Viral constructs production and purification

cDNA of 2MT-GCaMP6m (mitoGCaMP6m) was subcloned by restriction/ligation (restriction enzymes and T4-ligase were from Fermentas/Thermo Scientific Life Science Research) into a plasmid containing adeno-associated virus 2 (AAV2) inverted terminal repeats flanking a cassette consisting of the neuronal-specific human synapsin one promoter (hSyn), the woodchuck post-transcriptional regulatory element (WPRE) and the bovine growth hormone polyA signal.

Viral particles were produced in HEK293T cells (ATCC) as previously described (Tevet and Gitler, 2016), using pAdDelta5 helper plasmids (a kind gift from Dr. Adi Mizrahi) and the pAAV2/9 n plasmid (Addgene #112865) which encode the rep/cap proteins of AAV2 and AAV9, respectively. Viral particles were then purified over iodixanol (Sigma-Aldrich) step gradients and concentrated using Amicon filters (EMD). Virus titers were measured by determining the number of DNase I–resistant vector genomes (vg) using qPCR with a linearized genome plasmid as a standard (Challis et al., 2019).

### Stereotaxic injections

Mice at the age of P21-25 were deeply anesthetized with Ketamine/Xylazine and then stereotactic bilateral injections were performed into the Layer 5 of the somatosensory cortex using a microliter syringe (Hamilton, Israel) at a rate of 0.25 μl/minute, with 500 nl of AAV9-hSyn-Mito-GCaMP6m containing 1 × 10^10^ vg. After the injection, the needle was left in place for additional 3 min before being slowly removed from the brain. Coordinates for injections were (in mm): 4.1 rostral to lambda, ± 1.8 left/right of midline, –0.5 ventral to the pial surface.

### Acute coronal brain slices preparation

Coronal slices were prepared from mice three weeks post-injection (at the age of 6–7 weeks). The 300-µm-thick coronal cortical or horizontal hippocampal slices were prepared using standard techniques, as previously described (Baranauskas et al., 2013; Fleidervish et al., 2010). Mice were anesthetized with isoflurane (5%) and decapitated. The slices were cut on a vibratome (VT1200; Leica) and placed in a holding chamber containing oxygenated artificial cerebrospinal fluid (ACSF) at room temperature; they were transferred to a recording chamber after more than 1 hr of incubation. The composition of the ACSF (in mM): 124 NaCl, 3 KCl, 2 CaCl_2_, 2 MgSO_4_, 1.25 NaH_2_PO_4_, 26 NaHCO_3_, and 10 glucose; pH 7.4 when saturated with 95% O_2_/CO_2_.

### Fluorescence imaging

Most experiments were performed on L5 pyramidal neurons in somatosensory neocortical slices. The cells were viewed with a 40 or 60× Olympus water-immersion lens of Ultima IV two-photon microscope (Bruker) equipped with a Mai Tai Deep See pulsed laser (Spectra-Physics). MitoGCaMP6m was excited at 940–950 nm. L5 pyramidal cells with low resting fluorescence that responded to electrical stimulation delivered via the nearby placed bipolar electrode were selected for whole cell recording (see below). In order to measure the cytosolic Ca^2+^ transients, the intracellular solution was supplemented by Ca^2+^ indicator, Fura-2 (100 µmol/l). The indicator was selected to minimize the interference with the mitoGCaMP6m fluorescence measurements. The Fura-2 fluorescence was elicited by two-photon excitation at 780 nm, and it declined as a function of the cytosolic Ca^2+^ concentration. The neuronal morphology and the labelled mitochondria localization was obtained by scanning a Z-series of 30–40 high-resolution images at interval of 0.5 µm. The dynamic mitoGCaMP6m and Fura-2 imaging were performed from small regions of interest at frame rate of 10–50 Hz.

### Electrophysiology

Somatic whole-cell recordings were obtained using patch pipettes pulled from thick-walled borosilicate glass capillaries (1.5 mm outer diameter; Science Products, Germany). All recordings were at 30°C ± 0.5°C maintained with a temperature control unit (Luigs & Neumann, Rattingen). For current-clamp experiments the pipette solution contained (in mM): 130 K–gluconate, 6 KCl, 2 MgCl_2_, 4 NaCl, and 10 Hepes, with pH adjusted to 7.25 with KOH. Pipettes had resistances of 5–7 MΩ when filled with this solution supplemented with Fura-2 (Molecular Probes). Recordings were made using a Multiclamp 700B amplifier (Molecular Devices) equipped with CV-7B headstage (Molecular Devices). Data were low–pass–filtered at 10 kHz (−3 dB), single-pole Bessel filter and digitized at 20 kHz using Digidata 1,322 A digitizer driven by PClamp 10 software (Molecular Devices). Care was taken to maintain the access resistance below 10 MΩ.

### Wide-field fluorescence imaging

The NAD(P)H auto-fluorescence signals were obtained using a 40× water-immersion lens (Olympus) in a BX51WI microscope (Olympus). The fluorescence was excited by using a high-intensity LED device (385 ± 4 nm, Prizmatix), and the emission was collected by using a modified Olympus U-MNU2 filter set (DC = 400 nm; EM = 420 nm). Images were collected with the Orca Flash 4.0 CMOS camera (Hamamatsu), using a pixel binning of 512 × 512, at a rate of 300ms per frame. A bipolar stimulating electrode (WPI, 0.01 MΩ) was placed ~100 µM below the region of interest, at the L-5/6 border in cortical slices or in CA1 stratum oriens in the hippocampal slices. The 0.1ms long extracellular stimuli were delivered using an optically coupled stimulus isolation unit (A.M.P.I) driven via the pClamp 10 software. Somatic whole-cell recordings (see above) were made from a pyramidal neuron in the middle of the region of interest. The stimulation intensity was carefully controlled so that each stimulus triggered only a single antidromic spike with a latency of <1ms post-stimulus. The baseline fluorescence was kept around 1500 a.u. throughout the experiments by regulating the intensity of LED emitted light.

### Data analysis

Electrophysiological data analysis was accomplished using pCLAMP10 software (Molecular Devices) and Origin 6.0 (OriginLab). The figures were created using CorelDraw X7 suite (Corel Corporation).

### Statistical analysis

If not otherwise noted, data are expressed as mean ± SE. Student *t*-test for paired or unpaired data was used for statistical analysis.

## Data Availability

A representative subset of the raw electrical recording and imaging data has been deposited to Dryad (https://doi.org/10.5061/dryad.sxksn0348). The dataset contains the Microcal Origin opj files of the electrical and optical recordings and quantitative analysis of the data. We are unable to make all raw electrical recording and imaging data publicly available as due to the large size of our raw dataset (>10TB). Interested researchers should contact the corresponding author to gain access to the raw data. The following dataset was generated: FleidervishI StolerO StavskyA KhrapunskyY MelamedI StutzmannG GitlerD SeklerI 2022Frequency- and spike-timing-dependent mitochondrial Ca2+ signaling regulates the metabolic rate and synaptic efficacy in cortical neuronsDryad Digital Repository10.5061/dryad.sxksn0348PMC890680535192454

## Additional files

Transparent reporting form

## References

[bib1] Ashrafi G, de Juan-Sanz J, Farrell RJ, Ryan TA (2020). Molecular Tuning of the Axonal Mitochondrial Ca^2+^ Uniporter Ensures Metabolic Flexibility of Neurotransmission. Neuron.

[bib2] Attwell D, Laughlin SB (2001). An energy budget for signaling in the grey matter of the brain. Journal of Cerebral Blood Flow and Metabolism.

[bib3] Baranauskas G, David Y, Fleidervish IA (2013). Spatial mismatch between the Na+ flux and spike initiation in axon initial segment. PNAS.

[bib4] Baughman JM, Perocchi F, Girgis HS, Plovanich M, Belcher-Timme CA, Sancak Y, Bao XR, Strittmatter L, Goldberger O, Bogorad RL, Koteliansky V, Mootha VK (2011). Integrative genomics identifies MCU as an essential component of the mitochondrial calcium uniporter. Nature.

[bib5] Bi GQ, Poo MM (1998). Synaptic modifications in cultured hippocampal neurons: dependence on spike timing, synaptic strength, and postsynaptic cell type. The Journal of Neuroscience.

[bib6] Brecht M, Sakmann B (2002). ‐Dynamic representation of whisker deflection by synaptic potentials in spiny stellate and pyramidal cells in the barrels and septa of layer 4 rat somatosensory cortex. The Journal of Physiology.

[bib7] Celsi F, Pizzo P, Brini M, Leo S, Fotino C, Pinton P, Rizzuto R (2009). Mitochondria, calcium and cell death: A deadly triad in neurodegeneration. Biochimica et Biophysica Acta (BBA) - Bioenergetics.

[bib8] Challis RC, Ravindra Kumar S, Chan KY, Challis C, Beadle K, Jang MJ, Kim HM, Rajendran PS, Tompkins JD, Shivkumar K, Deverman BE, Gradinaru V (2019). Systemic AAV vectors for widespread and targeted gene delivery in rodents. Nature Protocols.

[bib9] Connors BW, Prince DA (1982). Effects of local anesthetic QX-314 on the membrane properties of hippocampal pyramidal neurons. The Journal of Pharmacology and Experimental Therapeutics.

[bib10] De Stefani D, Raffaello A, Teardo E, Szabò I, Rizzuto R (2011). A forty-kilodalton protein of the inner membrane is the mitochondrial calcium uniporter. Nature.

[bib11] De Stefani D, Rizzuto R, Pozzan T (2016). Enjoy the Trip: Calcium in Mitochondria Back and Forth. Annual Review of Biochemistry.

[bib12] Devaraju P, Yu J, Eddins D, Mellado-Lagarde MM, Earls LR, Westmoreland JJ, Quarato G, Green DR, Zakharenko SS (2017). Haploinsufficiency of the 22q11.2 microdeletion gene Mrpl40 disrupts short-term synaptic plasticity and working memory through dysregulation of mitochondrial calcium. Molecular Psychiatry.

[bib13] Díaz-García CM, Meyer DJ, Nathwani N, Rahman M, Martínez-François JR, Yellen G (2021). The distinct roles of calcium in rapid control of neuronal glycolysis and the tricarboxylic acid cycle. eLife.

[bib14] Divakaruni SS, Van Dyke AM, Chandra R, LeGates TA, Contreras M, Dharmasri PA, Higgs HN, Lobo MK, Thompson SM, Blanpied TA (2018). Long-Term Potentiation Requires a Rapid Burst of Dendritic Mitochondrial Fission during Induction. Neuron.

[bib15] Fleidervish IA, Lasser-Ross N, Gutnick MJ, Ross WN (2010). Na+ imaging reveals little difference in action potential-evoked Na+ influx between axon and soma. Nature Neuroscience.

[bib16] Gazit N, Vertkin I, Shapira I, Helm M, Slomowitz E, Sheiba M, Mor Y, Rizzoli S, Slutsky I (2016). IGF-1 Receptor Differentially Regulates Spontaneous and Evoked Transmission via Mitochondria at Hippocampal Synapses. Neuron.

[bib17] Giorgio V, von Stockum S, Antoniel M, Fabbro A, Fogolari F, Forte M, Glick GD, Petronilli V, Zoratti M, Szabó I, Lippe G, Bernardi P (2013). Dimers of mitochondrial ATP synthase form the permeability transition pore. PNAS.

[bib18] Glancy B, Balaban RS (2012). Role of mitochondrial Ca2+ in the regulation of cellular energetics. Biochemistry.

[bib19] Holtmaat A, Svoboda K (2009). Experience-dependent structural synaptic plasticity in the mammalian brain. Nature Reviews. Neuroscience.

[bib20] Inglebert Y, Aljadeff J, Brunel N, Debanne D (2020). Synaptic plasticity rules with physiological calcium levels. PNAS.

[bib21] Kwon SK, Sando R, Lewis TL, Hirabayashi Y, Maximov A, Polleux F (2016). LKB1 Regulates Mitochondria-Dependent Presynaptic Calcium Clearance and Neurotransmitter Release Properties at Excitatory Synapses along Cortical Axons. PLOS Biology.

[bib22] Lewis TL, Kwon SK, Lee A, Shaw R, Polleux F (2018). MFF-dependent mitochondrial fission regulates presynaptic release and axon branching by limiting axonal mitochondria size. Nature Communications.

[bib23] Lin Y, Li LL, Nie W, Liu X, Adler A, Xiao C, Lu F, Wang L, Han H, Wang X, Gan WB, Cheng H (2019). Brain activity regulates loose coupling between mitochondrial and cytosolic Ca^2+^ transients. Nature Communications.

[bib24] Lisman J, Spruston N (2005). Postsynaptic depolarization requirements for LTP and LTD: a critique of spike timing-dependent plasticity. Nature Neuroscience.

[bib25] Mainen ZF, Malinow R, Svoboda K (1999). Synaptic calcium transients in single spines indicate that NMDA receptors are not saturated. Nature.

[bib26] Makinson CD, Tanaka BS, Sorokin JM, Wong JC, Christian CA, Goldin AL, Escayg A, Huguenard JR (2017). Regulation of Thalamic and Cortical Network Synchrony by Scn8a. Neuron.

[bib27] Markram H, Lübke J, Frotscher M, Roth A, Sakmann B (1997a). Physiology and anatomy of synaptic connections between thick tufted pyramidal neurones in the developing rat neocortex. The Journal of Physiology.

[bib28] Markram H, Lübke J, Frotscher M, Sakmann B (1997b). Regulation of synaptic efficacy by coincidence of postsynaptic APs and EPSPs. Science.

[bib29] Miyazaki K, Ross WN (2017). Sodium Dynamics in Pyramidal Neuron Dendritic Spines: Synaptically Evoked Entry Predominantly through AMPA Receptors and Removal by Diffusion. The Journal of Neuroscience.

[bib30] Nowak L, Bregestovski P, Ascher P, Herbet A, Prochiantz A (1984). Magnesium gates glutamate-activated channels in mouse central neurones. Nature.

[bib31] Palty R, Silverman WF, Hershfinkel M, Caporale T, Sensi SL, Parnis J, Nolte C, Fishman D, Shoshan-Barmatz V, Herrmann S, Khananshvili D, Sekler I (2010). NCLX is an essential component of mitochondrial Na+/Ca2+ exchange. PNAS.

[bib32] Patron M, Granatiero V, Espino J, Rizzuto R, De Stefani D (2019). MICU3 is a tissue-specific enhancer of mitochondrial calcium uptake. Cell Death and Differentiation.

[bib33] Rangaraju V, Lauterbach M, Schuman EM (2019). Spatially Stable Mitochondrial Compartments Fuel Local Translation during Plasticity. Cell.

[bib34] Rizzuto R, Duchen MR, Pozzan T (2004). Flirting in little space: the ER/mitochondria Ca2+ liaison. Science’s STKE.

[bib35] Sjöström PJ, Turrigiano GG, Nelson SB (2001). Rate, timing, and cooperativity jointly determine cortical synaptic plasticity. Neuron.

[bib36] Spruston N, Schiller Y, Stuart G, Sakmann B (1995). Activity-dependent action potential invasion and calcium influx into hippocampal CA1 dendrites. Science.

[bib37] Svoboda K, Tank DW, Denk W (1996). Direct measurement of coupling between dendritic spines and shafts. Science (New York, N.Y.).

[bib38] Szabadkai G, Duchen MR (2008). Mitochondria: the hub of cellular Ca2+ signaling. Physiology (Bethesda, Md.).

[bib39] Tevet Y, Gitler D (2016). Using FRAP or FRAPA to Visualize the Movement of Fluorescently Labeled Proteins or Cellular Organelles in Live Cultured Neurons Transformed with Adeno-Associated Viruses. Methods in Molecular Biology (Clifton, N.J.).

[bib40] Wescott AP, Kao JPY, Lederer WJ, Boyman L (2019). Voltage-energized Calcium-sensitive ATP Production by Mitochondria. Nature Metabolism.

[bib41] Zott B, Simon MM, Hong W, Unger F, Chen-Engerer HJ, Frosch MP, Sakmann B, Walsh DM, Konnerth A (2019). A vicious cycle of β amyloid-dependent neuronal hyperactivation. Science (New York, N.Y.).

